# Acoustic Forward Model for Guided Wave Propagation and Scattering in a Pipe Bend

**DOI:** 10.3390/s22020486

**Published:** 2022-01-09

**Authors:** Carlos-Omar Rasgado-Moreno, Marek Rist, Raul Land, Madis Ratassepp

**Affiliations:** 1 Department of Civil Engineering and Architecture, Tallinn University of Technology, 19086 Tallinn, Estonia; 2 Thomas Johann Seebeck Department of Electronics, Tallinn University of Technology, 19086 Tallinn, Estonia; marek.rist@taltech.ee (M.R.); raul.land@taltech.ee (R.L.)

**Keywords:** guided waves, scattering, pipe bend, acoustic model, Thomsen parameters, finite differences

## Abstract

The sections of pipe bends are hot spots for wall thinning due to accelerated corrosion by fluid flow. Conventionally, the thickness of a bend wall is evaluated by local point-by-point ultrasonic measurement, which is slow and costly. Guided wave tomography is an attractive method that enables the monitoring of a whole bend area by processing the waves excited and received by transducer arrays. The main challenge associated with the tomography of the bend is the development of an appropriate forward model, which should simply and efficiently handle the wave propagation in a complex bend model. In this study, we developed a two-dimensional (2D) acoustic forward model to replace the complex three-dimensional (3D) bend domain with a rectangular domain that is made artificially anisotropic by using Thomsen parameters. Thomsen parameters allow the consideration of the directional dependence of the velocity of the wave in the model. Good agreement was found between predictions and experiments performed on a 220 mm diameter (d) pipe with 1.5d bend radius, including the wave-field focusing effect and the steering effect of scattered wave-fields from defects.

## 1. Introduction

Industrial pipelines are largely used to transport highly volatile fluids such as petrochemical products, steam, gas, or oil. However, these pipelines are vulnerable to corrosion and erosion damage [1,2]. Even a pipeline operating with minimal defects can experience catastrophic failures, such as the explosion of the vinyl petrochemical plant Pajaritos in the Gulf of Mexico, which was caused by a leak in one of the principal pipes [3]. Corrosion is often found in fittings, features, crossings, and complex geometries such as bends [4]. Particularly, the sections of pipe bends are damage hot spots due to the sudden change in the fluid flow direction and velocity, thus leading to significant wall thinning by flow-accelerated corrosion [5]. Erosion occurs when there are particles in the flowing solution damaging both the surface layers and the base metal. Therefore, detecting and quantifying damage in critical parts is crucial to guaranteeing the proper operation of pipelines and avoiding disastrous failures.

Common nondestructive testing techniques for pipe bends include local thickness gauges, radiography, and in-pipe robots. Local thickness gauges are handheld measuring devices capable of determining the thickness of a given material using mostly the travel time of ultrasound propagating through the thickness [6]. Although gauges are portable and small training is required to operate them, it is a slow method to cover large areas of interest. Radiography methods are based on using X-rays to penetrate a given structure. Even though they are quite sensitive, measurement systems are bulky, and users are exposed to the X-rays [7]. In addition, in-pipe robots are able to inspect curved pipelines and quantify damages in the structure [8]. Nonetheless, the main drawback is that the inspected pipe must be removed from operation in order to place the in-pipe robot for inspection and maintenance.

In contrast, ultrasonic guided waves (GWs) have been demonstrated to be an efficient tool for structural health monitoring [9,10,11]. They are capable of covering large distances in pipelines, being sensitive to cracks and corrosion [12,13,14]. Usually, ordinary pipeline screening is performed by a single transducer array attached to the pipe in a ring form. The array consists of a fixed number of transducers that can send out the required signals and receive the reflections from the potential defects. As a result, GWs are suitable for constant monitoring, damage detection, developing proper maintenance cycles, and predicting the remaining life and service of pipelines, including bends [15,16,17,18,19]. However, GW propagation in a pipe elbow is more complicated than in a straight pipe due to the curvature of the bend, and it has been studied by a number of authors. For example, Rose et al. [20] reported the natural focusing effect of GWs in a pipe bend and analyzed the echo waveforms for sensing a drill defect. Demma et al. [21] and Hayashi et al. [22] demonstrated that the reflection and transmission of guided waves in the bend are affected by the mode conversions. Rudd et al. [23] studied GW propagation around bends using the elastodynamic finite integration technique. Sanderson et al. [24] introduced an analytical method for GW propagation in the bend and studied the transmission of the T(0,1) mode. Heinlein et al. [25] investigated the reflection of the T(0,1) mode from the cracks in a bend. Xu et al. [26] studied the relationship of GW reflection amplitude with the angle of the bend using a guided wave denoising method. Overall, the primary limitation of the existing screening approaches in characterizing defects in bends is that the information about the defect is limited and therefore does not allow mapping the defect’s profile or properly monitoring its progression.

**Figure 1:**
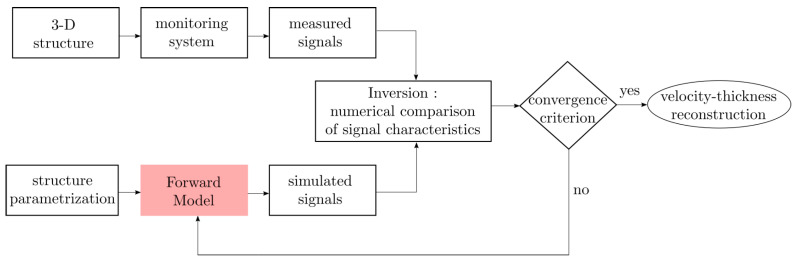
Flow chart of the GWT algorithm: real data (measured signals) and synthetic data (from forward model) are compared iteratively until a given residual criteria is reached. The role of the forward model is highlighted.

An alternative to overcoming the limitations of ordinary screening is to combine the methods with tomographic techniques. To do so, two transducer rings can be attached to the pipe instead of one, to make it possible to obtain a set of measurements from different angles. Guided wave tomography (GWT) works under the principle of measuring waveforms to form a wall thickness map of a given section. Then, any variation in the waveforms can be interpreted by tomographic algorithms as a thickness reduction in a specific location [27]. The reconstruction of the defects in GWT is based on the solution of an inverse problem that uses a forward model to predict a synthetic dataset for a given structure and a defect shape. The shape of the defect is updated iteratively by minimizing the residuals between the true and synthetic measurements until a convergence criterion is reached. Then, an accurate thickness reconstruction is obtained. The flow chart of the GWT algorithm is shown in Figure 1, where the role of the forward model is highlighted. As a result, the accuracy of the method is strongly influenced by a forward model capable of describing the guided wave propagation in a given structure [28].

In general, approximate 2D forward models are preferred over exact 3D models, due to their simplicity, low computational cost, feasibility of combining with tomographic algorithms, and their capability to evaluate the thickness of the waveguides from the velocity. Furthermore, the majority of research on GWT has focused on simple geometries, such as flat plates [29,30,31,32] and straight pipes [33,34,35]. However, only a few studies have investigated the application of GWT to pipe bends. Volker and van Zon [36] developed a forward model based on a recursive wave-field extrapolation and a deformed 2D planar grid and applied it in travel-time tomography. Brath et al. [37] introduced a 2D rectangular forward model for GW propagation in the bend, in which the equivalence was established by the travel-time-preserving orthogonal parametric presentation of the bend. Later, it was extended to curved ray tomography [38]. A similar model with the sparse inversion method was used by Wang and Li [39] in GWT for quantifying defects in the bend. However, all these approaches are limited to using the travel times of first arrivals, which limits the resolution of tomographic imaging [40].

In this study, we developed a 2D acoustic forward model for pipe bends that can be further used in tomography along with the full waveform inversion (FWI) algorithm. The FWI makes use of the full information of the wave field, thus enabling more accurate inversion results to be achieved compared to travel-time tomography [41]. The forward model is discretized by the finite difference (FD) method and the equivalence with the bend domain is established by an artificial anisotropic formulation using Thomsen parameters [42]. We investigated the transmission of the $A_{0}$ mode through a 90° pipe bend from different excitation points and compared the simulation results of the FD method with the results obtained from finite element (FE) modeling and experiments. In addition, the scattering from artificial defects in the bend was analyzed.

This paper is structured as follows: Section 2 outlines the methods for translating the 3D geometry of the bend into the 2D domain, and the implemented numerical methods are presented in Section 3. This approach is subsequently validated with the experimental setup described in Section 4, and the results are presented in Section 5. Finally, conclusions are drawn in Section 6.

## 2. Forward Model

In this section, we describe the equivalent 2D wave propagation model for the simulation of guided waves in a 3D pipe bend. Firstly, the 3D modeling domain is mapped to the 2D domain by using orthogonal parameterization of the space. Secondly, the anisotropic wave propagation model is introduced and then anisotropic Thomsen parameters are defined to fit the acoustic velocity model with the real guided wave model.

### 2.1. Orthogonal Parameterization

**Figure 2:**
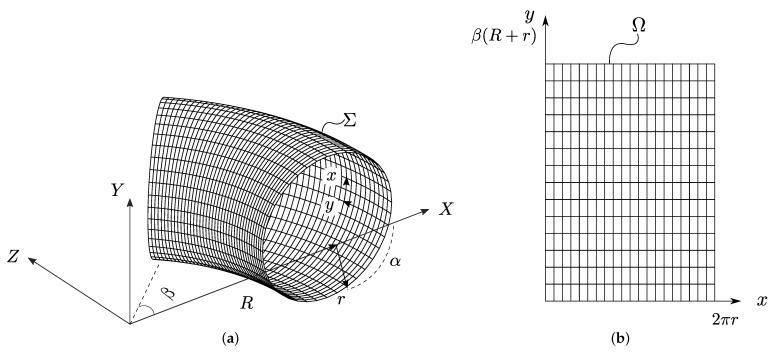
A torus section representing the bend of a pipe is translated from (**a**) the 3D space domain Σ to (**b**) the 2D space domain Ω.

Consider the section of the torus in Figure 2a, defined in the 3D space domain Σ, with mid-thickness or central radius *r*, bend radius *R*, torus azimuth longitude β around the *Y*-axis, and torus latitude α with respect to the XY plane. To model the anisotropic wave propagation around the bend in the 2D space domain Ω shown in Figure 2b, a translation of the torus section from Σ to Ω is needed. We followed the parametrization proposed by Brath et al. [37]. According to this, the torus section is unwrapped from the longest radius path of the bend (at extrados position R+r). Secondly, the 2D horizontal and vertical axes *x* and *y* were set equal to the torus circumference 2πr and the bend’s extrados azimuth length β(R+r), respectively. As a result, the 2D domain Ω can be expressed as Ω=[0,2πr]×[0,β(R+r)], with the extrados position located at the middle of the circumference at (πr,y). The intrados position is located at the beginning of the circumference at (0,y) and at the end (2πr,y).

Orthogonal parameterization of the torus is given by the set of equations as follows:

(1) $$\begin{array}{ccc} X & = & \left(R+r\cos\frac{x}{r}\right)\cos\frac{y}{R+r}\\ Y & = & r\sin\frac{x}{r}\\ Z & = & \left(R+r\cos\frac{x}{r}\right)\sin\frac{y}{R+r}, \end{array}$$

where the two-dimensional coordinates {x,y} are used to express the three-dimensional space coordinates {X,Y,Z}. In this way, the 3D space coordinates can be mapped directly into the 2D domain, and vice versa.

### 2.2. Acoustic Wave Equation

**Figure 3:**
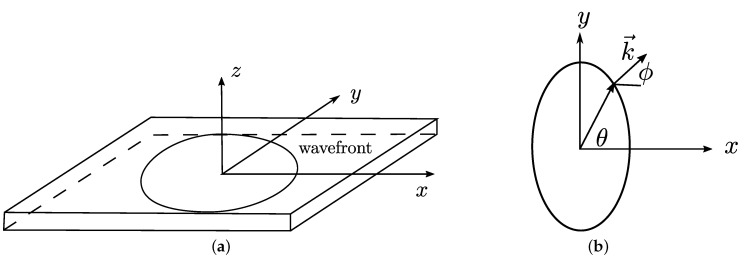
(**a**) Acoustic model of GW propagation in a plate with vertical transverse isotropy. (**b**) Top view of the wave field, group angle θ, wave number $\vec{k}$, and phase angle ϕ.

We assume that a guided wave propagating in a bent wall of varying thickness will behave the same as an acoustic wave traveling in a 2D medium with varying velocity. The model is shown in Figure 3, and the acoustic wave is modeled in transversely isotropic media with a vertical symmetry axis and is described by fourth-order partial differential equations in time *t* [43]:

(2) $$\frac{\partial^{4}F}{\partial t^{4}}-v_{\phi }^{2}\left(1+2\eta \right)\left(\frac{\partial^{4}F}{\partial y^{2}\partial t^{2}}\right)=v_{0}^{2}\frac{\partial^{4}F}{\partial x^{2}\partial t^{2}}-2\eta v_{0}^{2}v_{\phi }^{2}\left(\frac{\partial^{4}F}{\partial y^{2}\partial x^{2}}\right)$$

with η defined as

$$\eta =\frac{\epsilon -\delta }{1+2\delta }.$$

In Equation (2), F(x,y,t) describes the pressure field of the propagating wave, $v_{\phi }$ is the phase velocity, $v_{0}$ is the phase velocity along the *x*-axis, and ϵ,δ is the nondimensional Thomsen parameters to describe the anisotropy of the wave field.

### 2.3. Implementation for a Pipe Bend

To link the phase velocity $v_{\phi }$ of the acoustic wave to the geometry of the pipe, we use the approximated Thomsen parameters [42,44], which are defined by the phase velocity $v_{\phi }$ at three different angles, horizontal $v_{0}$, vertical $v_{\frac{\pi }{2}}$, and $v_{\frac{\pi }{4}}$:

(3) $$\begin{array}{ccc} \epsilon & = & \frac{v_{\frac{\pi }{2}}-v_{0}}{v_{0}}\\ \delta & = & 4\left(\frac{v_{\frac{\pi }{4}}}{v_{0}}-1\right)-\epsilon , \end{array}$$

where ϵ quantifies the velocity difference between the wave propagation along the vertical direction, and δ is the wave propagation at intermediate phase angles.

As the pipe bend is unwrapped at the extrados position, the velocities $v_{\frac{\pi }{2}}=v_{0}$ and ϵ=0. Additionally, the vertical velocity $v_{\frac{\pi }{2}}$ at each point of the circumference can be expressed in terms of distance as $\left(R+r\cos\left(\alpha \right)\right)/\left(R+r\right)v_{0}$. In this way, ϵ can be written as a function of the radius of the bend *R* and the central radius *r* of the pipe as

(4) $$\epsilon =\frac{R+r}{R+r\cos\alpha }-1.$$

In addition, the phase velocity $v_{\frac{\pi }{4}}$ used to obtain δ can be computed from the phase angle ϕ related to the group angle θ [37] described by

(5) $$\begin{array}{ccc} \tan\phi & = & \frac{\tan\theta }{{\left(\frac{v_{\pi /2}}{v_{0}}\right)}^{2}}. \end{array}$$

In this way, we use Equations (3) and (4) to compute Thomsen parameters for GW propagation in the bend.

## 3. Numerical Methods

### 3.1. Configuration of the Problem

**Figure 4:**
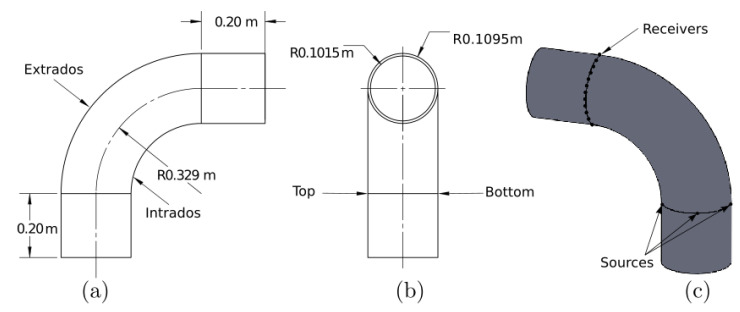
Schematic of the pipe bend. (**a**) Top view, (**b**) lateral view, and (**c**) isometric view of the simulated pipe bend.

For both models and measurements, we considered the steel pipe specimen described in Figure 4, with inner radius $r_{in}=0.1015$ m, outer radius $r_{out}=0.1095$, 90° bend with radius R=0.329 m, and two straight pipe sections at the beginning and at the end of the bend that are 0.20 m long each (only included in the 3D model as absorbing regions). The steel’s properties are listed in Table 1. We refer to the longest and shortest arcs of the bend as extrados and intrados, respectively. Similarly, we use top and bottom for the mid-bend’s arc between the intrados and extrados.

Guided waves were excited at the beginning of the bend at three source positions: intrados, top, and extrados. We expected that the waves excited from these points would propagate differently due to the geometric anisotropy that can be described by Thomsen parameters. For the excitation, we used a five-cycle $A_{0}$ mode with central frequency at 50 kHz modulated by a Hanning window. The waves were monitored at the other side of the bend at 20 receiving points that were equally distributed around the circumference.

**Figure 5:**
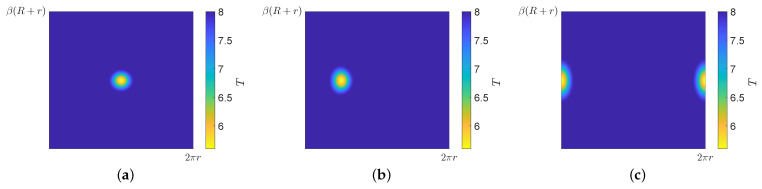
Thickness *T* (mm) in the 2D acoustic domain with a Hann-shaped defect at three different locations: (**a**) extrados, (**b**) top, and (**c**) intrados.

To investigate the scattering of the waves from defects, we modeled a Hann-shaped thickness reduction defect with a center $\left\{X_{c},Y_{c},Z_{c}\right\}$ by resizing the pipe thickness *T* from the outer surface using the following equation:

(6) $$T=\left\{\begin{array}{cc} t_{0}-\frac{D}{2}\left[1+\cos\left(\frac{2\pi |T|}{W}\right)\right], & \;|T|<\frac{W}{2}\\ t_{0}, & |T|>\frac{W}{2} \end{array}\right.$$

where $\left|T\right|=\sqrt{{\left(X-X_{c}\right)}^{2}+{\left(Y-Y_{c}\right)}^{2}+{\left(Z-Z_{c}\right)}^{2}}$, $t_{0}$ is the nominal pipe thickness, and *D* and *W* are the depth and width of the defect, respectively. In this study, the size of the selected defect was 120 mm wide and its maximum depth was 30% of the thickness reduction. The defect was placed at the center of the pipe elbow and was simulated in three locations: extrados, top, and intrados. The respective 2D thickness models are shown in Figure 5. It can be seen that the defect was circular at the extrados and became elliptical when moving toward the intrados as a consequence of the unwrapping of the bend.

### 3.2. FE Modeling

**Figure 6:**
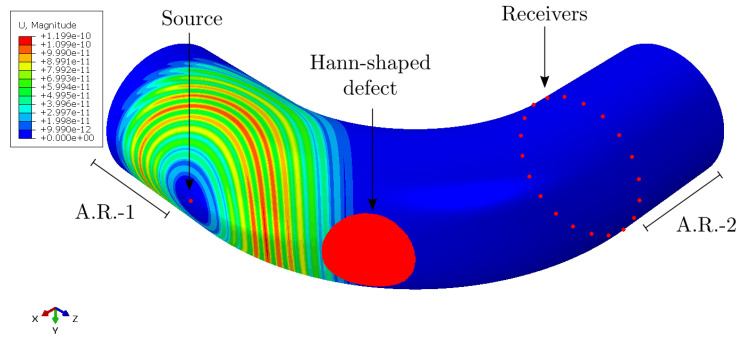
Geometry of the pipe bend simulated in ABAQUS for the *extrados* case with a Hann-shaped defect, absorbing regions (ARs) 1 and 2, and displacements of the propagating waves shown at 100 µs.

The ABAQUS Explicit software was used [45] for the 3D guided wave propagation simulations in the pipe bend described in Figure 4. To build the section of the bend, first, we defined a circular mesh with 560 elements along the circumference of the pipe and 6 elements along with the thickness. Second, we rotated the circular mesh according to the geometry of the pipe bend, as shown in Figure 4a. The number of elements along the 90 degree rotation was 550. The eight-node brick element type C3D8R was used. In order to avoid reflections from the boundaries, we defined absorbing regions on the straight pipe sections [46]. The number of elements along each straight section was 160. On one bend’s side, we generated the $A_{0}$ mode by applying an out-of-plane force in the radial direction. We excited three different source positions separately as we described previously (intrados, top, and extrados positions). On the other bend’s side, we measured radial displacement components. A sample of the pipe bend geometry simulated in ABAQUS for the extrados case with a Hann-shaped defect is shown in Figure 6.

In addition to transmission measurements, the scattering from Hann-shaped defects was investigated. The defect wave field was isolated by using the baseline wave-field subtraction.

### 3.3. Acoustic Modeling

**Figure 7:**
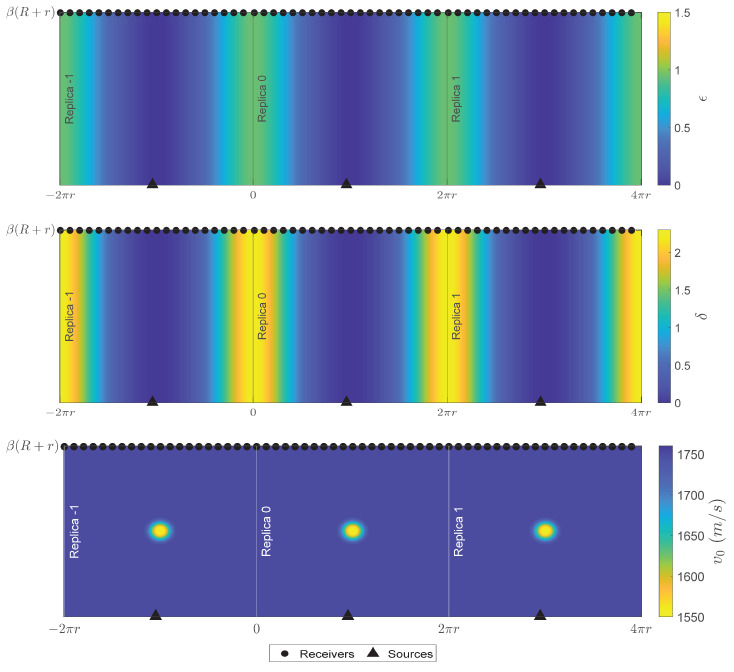
(**a**,**b**) *ϵ* and *δ* distribution along the bend with 3 replicas, respectively. Sources were placed at the extrados position of each replica. (**c**) $v_{0}$ in the bend with a Hann-shaped defect located at the extrados position. A similar velocity model was created for each computed frequency.

The simulations in the acoustic domain were performed with the finite difference (FD) method using the mixed-grid approach [47]. The calculation domain consisted of three replicas to include the higher-order helical wave paths in the simulation [37]. Each replica was discretized with 161 grid points along the circumferential direction and 166 grid points in the axial direction, and it was sampled with a grid step of 4.143 mm. The background models showing the distribution of Thomsen parameters and the velocity distribution of the bend with the defect and the source located at the extrados position are shown in Figure 7. The wave propagation was isotropic along the extrados of the bend where Thomsen parameters were zero, while it became increasingly anisotropic when the waves transmitted through the intrados area where Thomsen parameters are the largest. The velocity models at a required frequency were obtained from the thickness map by using the velocity–frequency–thickness dispersion curve of the $A_{0}$ mode. To obtain the higher-order helical wave paths in the simulation, the source in each replica was excited separately and their responses were summed. The procedure was repeated with the source and the defect located at the top and intrados position of the bend.

Calculations were performed using the two-dimensional frequency-domain engine TOY2DAC [48] to solve Equation (2) in the frequency domain for the required frequency components. The inverse fast Fourier transform was used to transform the frequency-domain results into a time domain. A 2x Intel Xeon E5-2660v2, 64 GB RAM cluster was used for the computation, and the calculation time was 7 min for a single excitation case.

## 4. Experimental Measurements

**Figure 8:**
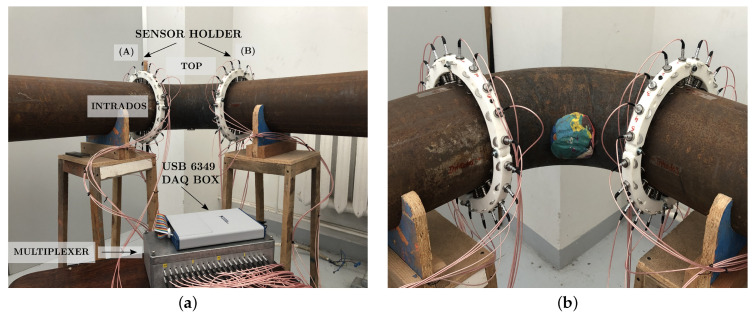
(**a**) Steel pipe bend specimen with the same characteristics as in Table 1. Measurement setup with transducer arrays A and B with 20 transducers each and an acquisition system; (**b**) pipe bend with an artificial defect composed of plasticine and located on the intrados position, *intrados* view.

We used the experimental setup shown in Figure 8a. It consisted of a steel pipe bend, which was supported by four wooden holds, and it had the same properties and dimensions as described in Table 1 and Figure 4a, respectively; the only exception was that the straight pipe sections were 1 m long each, so that the reflections with the edges were avoided in the measurement. The measurement setup consisted of two rings of transducers located at each end of the bend, one multiplexer for exciting the desired signal, and a data acquisition box.

Each ring of transducers (ring A and ring B) contained 20 piezoelectric transducers (Doppler Ltd., Guangzhou, China) with a central frequency of 50 kHz. They were used as both transducers and receivers. The transducers were equally distributed along the circumference and they were pressed against the pipe with springs so that the excitation was applied in the radial direction. Similarly, radial displacements were measured with the receiving transducer ring. Sensor no. 1 was located at the intrados, sensor no. 6 was at the top, and sensor no. 11 was at the extrados. The used multiplexer contained 20 sensing channels for amplification and conditioning the measured responses. The multiplexer network allowed switching the excitation to any transducer at one ring, and the receiving channels were connected to all the elements on the opposite ring.

In addition, the multifunctional USB-6349 from National Instruments was used for data acquisition. It was connected to the PC via a USB and featured 32 simultaneous analog input channels with a 500 kS/s sampling rate and 16-bit resolution. It also contained two 16-bit analog output channels and 24 general-purpose digital input/output channels. The acquisition software was created in LabView [49], and was used for creating the excitation waveforms, creating and running through the multiplexer channels, and connecting the piezoelectric elements to excitation amplifiers or receiving amplifiers. The excitation waveforms were sent to the data acquisition and the response waveforms were measured, digitally filtered, and logged.

The transmission of the $A_{0}$ mode through the bend was investigated for the three excitation points located at the extrados, top, and intrados positions of the bend. For the scattering studies, we used plasticine (ρ=1452.3 kg/m^3^, mass=0.244 kg, r=50 mm) attached to the surface of the pipe, as shown in Figure 8b. It is thought that the $A_{0}$ mode, which has a wave field dominated by the out-of-plane displacement, is sensitive to the coating layer and causes scattering. Additionally, plasticine has easy molding properties so it could be attached and removed from the pipe without changing the pipe’s material or the coupling condition between the transducers and the pipe. This was important for the baseline wave-field subtraction. Therefore, the position of the plasticine was changed for each excitation point and was aligned with the excitation point along the bend axis.

## 5. Results and Discussion

In this section, we investigate the wave field of the $A_{0}$ mode transmitted through the bend and its scattering from the defect located in the bend. The results from the experiment, FE, and FD modeling are presented and compared.

### 5.1. Guided Wave Propagation in the Pipe Bend

**Figure 9:**
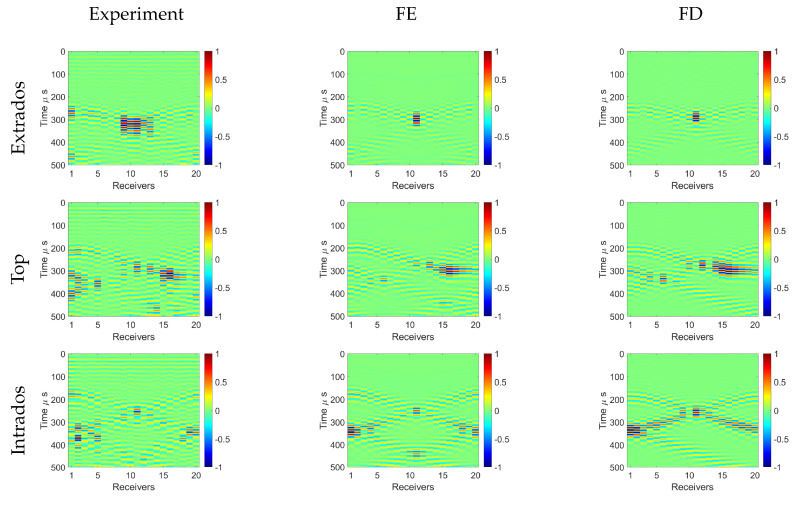
Experimental, FE, and FD contour plots of normalized radial displacement in the bend for three excitation positions: extrados, top, and intrados. The $A_{0}$ mode was exited with a central frequency of 50 kHz.

The $A_{0}$ mode at 50 kHz was excited separately in three different locations: at the extrados, top, and intrados of the bend beginning; and its propagating wave field was measured with a transducer ring at the bend end. The experimentally obtained contour plots of radial displacements of the received waves are shown in Figure 9 with FE and FD methods. The amplitudes of the received waves were normalized by the maximum displacement value of the recorded signals.

First, it can be seen that the recorded wave patterns differed for different excitation points, which occurred due to the effect of dissimilar ray paths and distances. A symmetric wave pattern can be seen in the case of the excitation located at the extrados and intrados positions, and nonsymmetric behavior was characteristic for the excitation at the top. This is related to the symmetry of the model parameters of the bend and the position of the excitation, as shown in Figure 7. Second, it can be observed that the direct first arrivals were followed by the wave-packets, which were helical waves propagating around the bend multiple times. Some helical waves arriving after 400 µs were not seen in the FD results due to the limited number of replicas used in the simulation.

Some specific observations were made for each excitation location. In the case of the extrados excitation, the slowest but most energetic signal was measured at the extrados position (transducer no. 11). This is due to the longest traveling distance along the extrados and the wave-field focusing caused by the lensing effect determined by the sound–speed valley [37]. When exciting from the top position (transducer no. 6), the waves naturally followed the shortest path toward the intrados and there were helical paths arriving before the wavefront. This was observed at receivers 13–20, where the focusing effect was also present but pushed more toward the intrados of the bend due to the horizontal velocity component of the wave. For the intrados excitation (transducer no. 1), the wave propagation was fastest along the intrados direction but lost more energy in this direction compared to the wavefront making the full circle around the bend. As the helical trajectory was also shorter compared to extrados excitation, the higher-order helical waves arrived earlier to the receivers. In addition, the right helical wave, which should have appeared at transducer 20 from 300 µs, was missing in the FD result. One extra replica is needed on the right side of the model to make it appear.

Overall, it can be seen that the FD results obtained by the proposed acoustic forward model agree well with the FE result, showing that the model can accurately represent reality and the experimental results. The results from the experiment were noisier and there were more variations in the amplitudes compared to the simulation results. This can be explained by the uneven coupling of the transducer to the pipe surface. In addition, there was notable noise present before 150 µs. This noise was a consequence of the crosstalk from the transducer source with the transducer receivers. However, the arrival times of the wave-packets well-matched the ones from the simulations, and we could observe the focusing effect in the case of extrados and top excitation. In general, this measurement example demonstrated the suitability of the introduced 2D acoustic model for predicting waveforms of guided waves in pipe bends.

### 5.2. Scattered Wave Fields in the Pipe Bend

The scattering of the $A_{0}$ mode was investigated from the defects at the center of the pipe elbow on the extrados, top, and intrados positions, separately. In simulations, a defect as the outer surface thickness reduced with Hann-shape variations was considered; in the experiment, a circular plasticine layer attached to the pipe surface was used. Wave fields scattered from the defect were isolated by subtracting the received waveforms of the intact pipe bend from the waveforms measured in the pipe bend with the defect.

**Figure 10:**
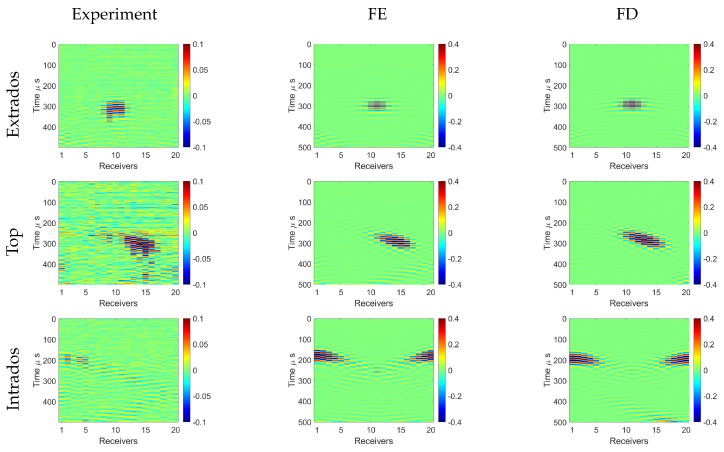
Experimental, FE, and FD scattered wave fields in the bend when the excitation sources and defects were located at three different positions: extrados, top, and intrados. Plasticine was used for producing scattered waves in the experiment, and a Hann-shaped defect was modeled in FE and FD simulations.

The contour plots of radial displacements of the scattered waves are shown in Figure 10, obtained experimentally with the FE and FD methods. The amplitudes of the waves were normalized by the maximum displacement value of the recorded signals from the intact pipe bend measurements. We focused on the first-arrived scattering, which was the most energetic. It can be seen that the scattering was dependent on the location of the excitation and the defect. In the case of the extrados excitation, we observed that the scattering was localized and remained at the extrados position. Interestingly, for the top excitation (transducer no. 6), the scattered wave was strongly steered and it arrived on the bottom side of the bend. Similar behavior to that of the waves was observed in the intact bend where the wave energy from the top excitation focused on the opposite side of the bend. Finally, when the excitation and the defect were at the intrados of the bend, the scattered wave followed the intrados direction but its pattern was much wider as the wave energy tried to escape from the sound-speed hill, which is opposite to the lensing effect and focusing.

In general, there was a clear resemblance in the scattered wave fields obtained with the different methods. The interaction of the wave with the plasticine in the experiment was a bit weaker compared to the interaction with the thickness reduction in the simulations. In addition, the experimental results were contaminated by noise, especially observed for the case with top excitation; however, the scattered wave field could be confidently observed. The biggest difference was observed with the intrados excitation, where the scattered field in the experiment was not symmetric compared to the modeling results, and spread across the bend. We think that this may have been caused by the directionality of the transducers, meaning that the waves were not equally excited around the intrados directions. In Figure 9, we can see that larger amplitudes were received at receivers 1–5 than those at 15–20, which could have resulted in some directivity in the scattering patterns. This scattering study demonstrated that the proposed acoustic model is capable of predicting correct waveforms from the defects in different locations, which is an important feature for accurate tomographic calculation.

## 6. Conclusions

In this study, an efficient and simple forward model was developed to simulate guided wave propagation and scattering in pipe bends. The 3D elastic bend was replaced by the 2D rectangular anisotropic acoustic domain by using orthogonal parameterization. The equivalence in the wave propagation was established based on implementing approximate anisotropic Thomsen parameters that describe the angular variability of the velocity in the acoustic model. The model was used to simulate the $A_{0}$ mode propagation and interaction with the defects from different excitation points across the bend. We found that the wave excited at the extrados focused at the extrados side of the bend. The wave excited from the top tended to focus on the opposite bottom side of the bend, which also caused the steering effect of the wave field scattered from the defect. The wave excited from the intrados tended to lose energy in other directions. Good agreement was found between the results from the proposed modeling method, the FE modeling, and the experimental results. Future work should involve combining the introduced forward model with FWI tomography to construct accurate corrosion maps for the pipe bends.

## Figures and Tables

**Table 1:** Steel pipe material properties. Density ρ, Young’s modulus *E*, and Poisson ratio ν.

ρ (kg/m^3^)	*E* (GPA)	ν
7932	216.9	0.2865

## Data Availability

The measurement data are available on request from the authors.
